# Characterization of the spontaneously recharging natural hydrogen reservoirs of Bourakebougou in Mali

**DOI:** 10.1038/s41598-023-38977-y

**Published:** 2023-07-22

**Authors:** Omar Maiga, Eric Deville, Jérome Laval, Alain Prinzhofer, Aliou Boubacar Diallo

**Affiliations:** 1 IFP-School, IFPEN, Rueil-Malmaison, France; 2 GEO4U, Rio de Janeiro, Brazil; 3 HYDROMA INC, Montréal, Québec Canada

**Keywords:** Hydrogen energy, Precambrian geology

## Abstract

In today’s race to find ways to produce cheap and green hydrogen, the natural hydrogen wells in Bourakebougou offer a promising solution and are a good example of how H_2_ can be produced in the natural environment. Not only has one well been successfully exploited to generate electricity for the local village, but twenty-four other exploratory boreholes have also demonstrated the presence of natural H_2_ in the surrounding area. The Bourakebougou H_2_ field offers a unique opportunity for geoscientists to determine the key characteristics of natural hydrogen reservoirs. This paper presents the coring, logging, and geochemistry studies that were performed to better characterize the nature of the Bourakebougou H_2_-bearing reservoirs. The shallowest main reservoir, in which there is the highest content of H_2_, is made of dolomitic carbonate (Neoproterozoic cap carbonate). These carbonates are largely karstified and show a high degree of heterogeneity in porosity (0.21–14.32%). Based on the analysis of the drilling imagery of the carbonated reservoirs, the accumulation of hydrogen occurs in the karst (void) representing a secondary porosity in the rock matrix. Other reservoirs, especially the deepest ones, are porous sandstone rocks with much more homogeneous porosities (4.52–6.37%) compared to the massive carbonates. For the wells analysed, the neutron tool reacted in a specific way when there is the presence of hydrogen. Hence, it stands out as being the primary tool to detect the presence of natural hydrogen beyond simple gas logging. When comparing a H_2_ reservoir system to classical oil and gas reservoir systems, the results show that the hydrogen reservoir is a dynamic system that is progressively recharged in H_2_-rich gas at the production timescale.

## Introduction

Over the past decade, natural hydrogen exploration has increased significantly on all continents^1–12^. The Bourakebougou field is the most advanced case with the most significant data available^7^. In production since 2012 to supply electricity to the village of Bourakebougou, the site is an emblematic example of an accumulation of natural hydrogen in an intracratonic continental context. It has therefore become important to use this case study to better understand what a natural hydrogen reservoir system is and to provide guidelines for further exploration. The Bourakebougou natural hydrogen in Mali was discovered during a water drilling operation in 1987. Since then, 24 other boreholes drilled by Hydroma (most of them at shallow depths) have discovered H_2_ accumulations as shallow as 100 m in several carbonate reservoirs covered by dolerite sills^7^. One of the 25 boreholes reached the basement and other deeper reservoirs have been identified within the Neoproterozoic sedimentary cover^7^. Gas Chromatography analysis of the gas phase in the shallower main reservoir has shown that the gas is mainly composed of natural H_2_ (98%) associated with nitrogen and methane (1% each). Gas logging has also shown a very high H_2_/CH_4_ ratio with generally higher values in the upper part of each individual reservoir^7^. The noble gases associated with this H_2_-rich gas have crustal signatures^13,7^. Old cratons and regions with iron-rich formations, especially the Neoproterozoic formations, are potentially prone for natural hydrogen generation^3,6,14^. In the case of the Bourakebougou field, Briere and Jerzykiewicz^15^ discussed 7 theories of H_2_ generation, but oxidation of Fe^II^-rich rocks associated to water reduction is assumed to be the main origin of H_2_. Today, whereas the presence of natural H_2_ appears to be increasingly common in the form of surface seepages, in particular on the continents but also in different geological contexts such as hydrothermal systems in mid-oceanic ridges^16–19^ and ophiolites^2,20–24^, we still do not know exactly what a natural hydrogen reservoir is and whether it can be compared with classical oil and gas reservoirs. The question that remains is what are the typical geological characteristics of an exploitable H_2_ reservoir? The objective of this study is to try to fill this knowledge gap. To do so, a detailed characterization of the H_2_-bearing reservoirs, using geological, mineralogical, and logging data was performed. This allowed us to better define the reservoirs’ properties, to better understand the accumulation process of H_2_ in the reservoirs, and to know what is the most efficient tool to characterize the presence of hydrogen, apart from a simple gas logging. The results obtained can be used as a reference to search for similar sites worldwide and to potentially identify target reservoirs for future productions.

## Geological context

**Figure 1:**
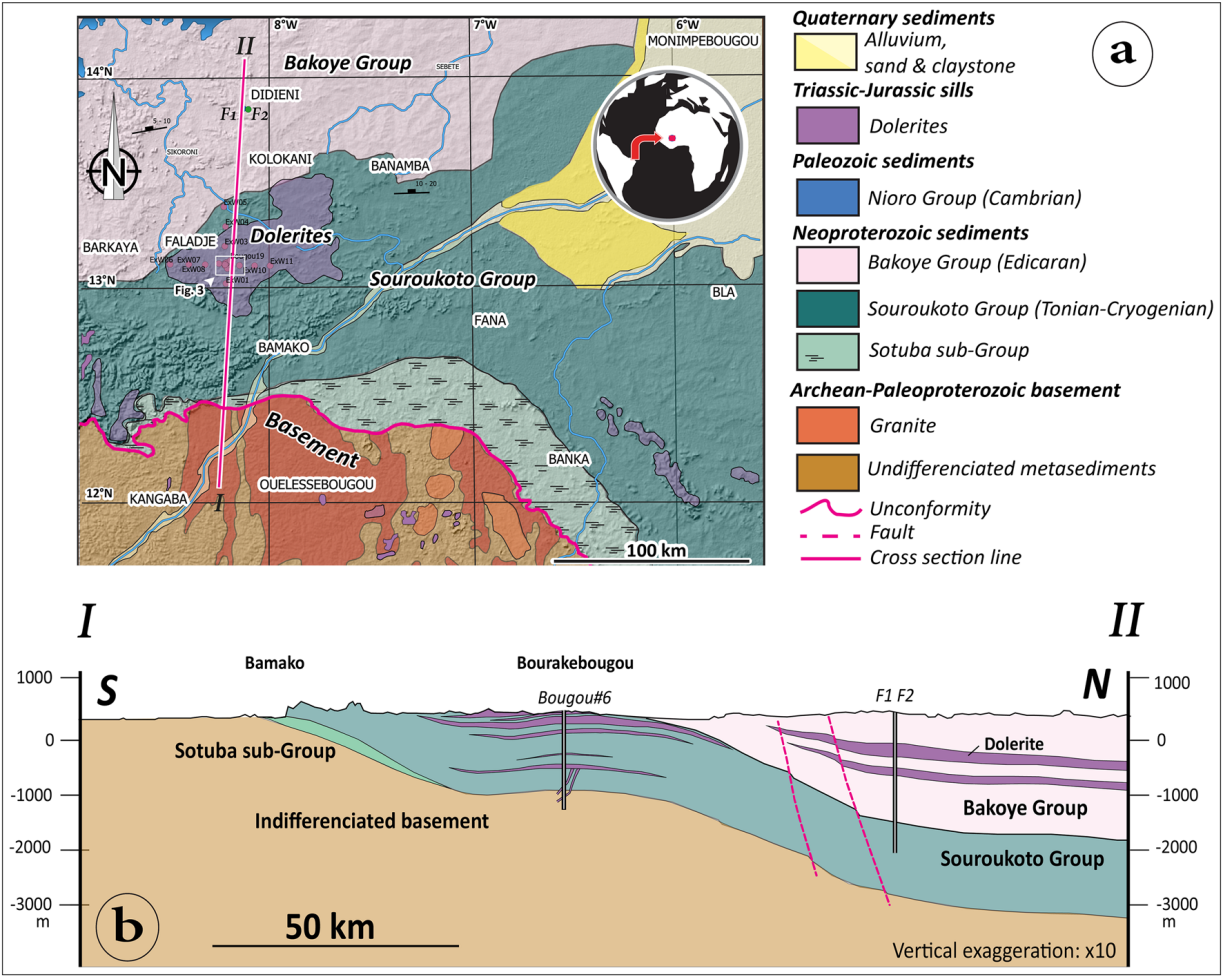
(**a**) Geological map of the study area designed and modified from the Global Geological Map of Mali (1/1 500 000), DNGM^29^, (**b**) Synthetic and simplified structural section of the study made according to the cross section line in Fig. 1(**a**). The maps were generated using Adobe illustrator CS6 (Version 16.0.0, https://www.adobe.com/).

**Figure 2:**
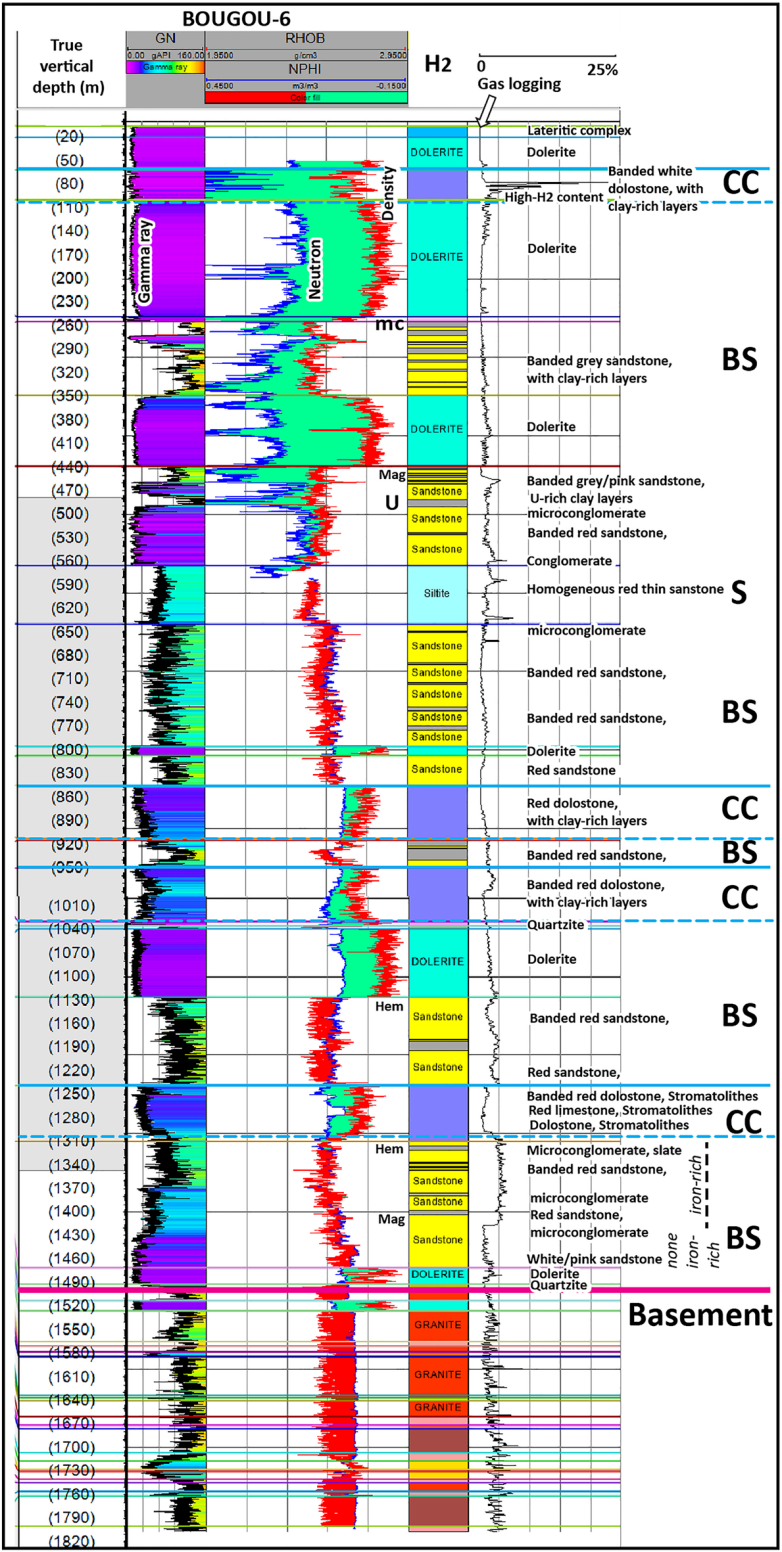
Synthetic stratigraphic log and electric logging of the Bougou#6 borehole, which is the most characteristic of the area studied in the Bourakebougou sector, from the surface to the basement. *CC* cap carbonate, *BS* banded sandstone, *S* massive sandstone, *mc* zone of intense contact metamorphism, *Mag* presence of magnetite, *U* uranium-rich level, *Hem* presence of hematite. The coloured lines correspond to the well tops.

The study area of the Bourakebougou field, is in the southern part of the Taoudeni Mega-Basin, in Mali (Fig. 1a). The formations found in this area cover a rather long time period, from the Archean to the Quaternary. The oldest formations, notably those corresponding to the basement (Fig. 1a and b), are located to the south of the study area and are part of the so-called Leo-Man Ridge. These formations are mainly composed of plutonic, volcano-sedimentary and sedimentary (Birrimian) rocks surrounded by a series of magmatic rocks that were affected by the Eburnean orogeny which took place between 2.2 and 2.0 Ga^25^. The basement rocks are essentially composed of granite, granodiorite, diorite, syenite and aplite. Above these basement formations lie unconformable sedimentary formations of Neoproterozoic age, characterized by sandstone-pelitic formations, carbonates and diamictites. Dolerite intrusions crosscut both the basement and the Neoproterozoic sediments forming mega-sills in the studied area (Fig. 1a and b). The Neoproterozoic sedimentary formations in the region are divided into two main Groups: The Bakoye Group in the North and the Souroukoto Group in the South. A sub-group can be distinguished within the Souroukoto Group: The Sotuba sub-group, known in the region for its richness in glauconite (Fig. 1a). The Bakoye Group has been characterized by Deynoux et al.^26^, Rossi et al.^27^ and Shields et al.^28^ as deposits controlled by glacial episodes. It includes essentially sandstone, diamictites and cap dolostone^26–28^. The Souroukoto Group and its sub-groups (Figs. 1a, 2**)** are mainly characterized by quartzites, sandstones with glauconite (fine and coarse to heterogranular), showing oblique stratifications at their base. Dolomites and diamictites resulting from Sturtian to Marinoan glacial episodes are also present. All the formations are intruded by doleritic sills attributed to the Triassic-Jurassic period (contemporary with the development of the Central Atlantic Magmatic Province), but this remains to be confirmed. Paleozoic ages are also possible.

On the simplified structural section of the area (Fig. 1b), a flexure of the basin with a depression towards the Taoudeni basin to the north can be observed. The F1 and F2 boreholes drilled north of the study area (Fig. 1a and b) did not reach the basement, even though their depths exceeded 2000 m. F1 and F2 essentially crossed the Bakoye Group (Fig. 1b). Dolerite sills affect most of the formations and form a dome in the Souroukoto Group that may contribute to trap hydrogen (Fig. 1a and b). On the Bourakebougou field, one of the 25 wells (Bougou-6) reached the basement. This well allowed us to make a stratigraphic log of the study area. The sediments correspond to the Souroukoto Group formations mentioned above and comprise banded sandstone-arkosic formations (Fig. 2). Some banded sandstone levels in the lower series of Bougou-6 shown in Fig. 2 are very rich in iron and can be considered as 'Banded Iron Formations' (BIF). The detrital levels alternate with dolomitic carbonates, sometimes brecciated, which can be interpreted as 'cap carbonates' (carbonates materializing the end of the glacial periods).

## Materials and methods

**Figure 3:**
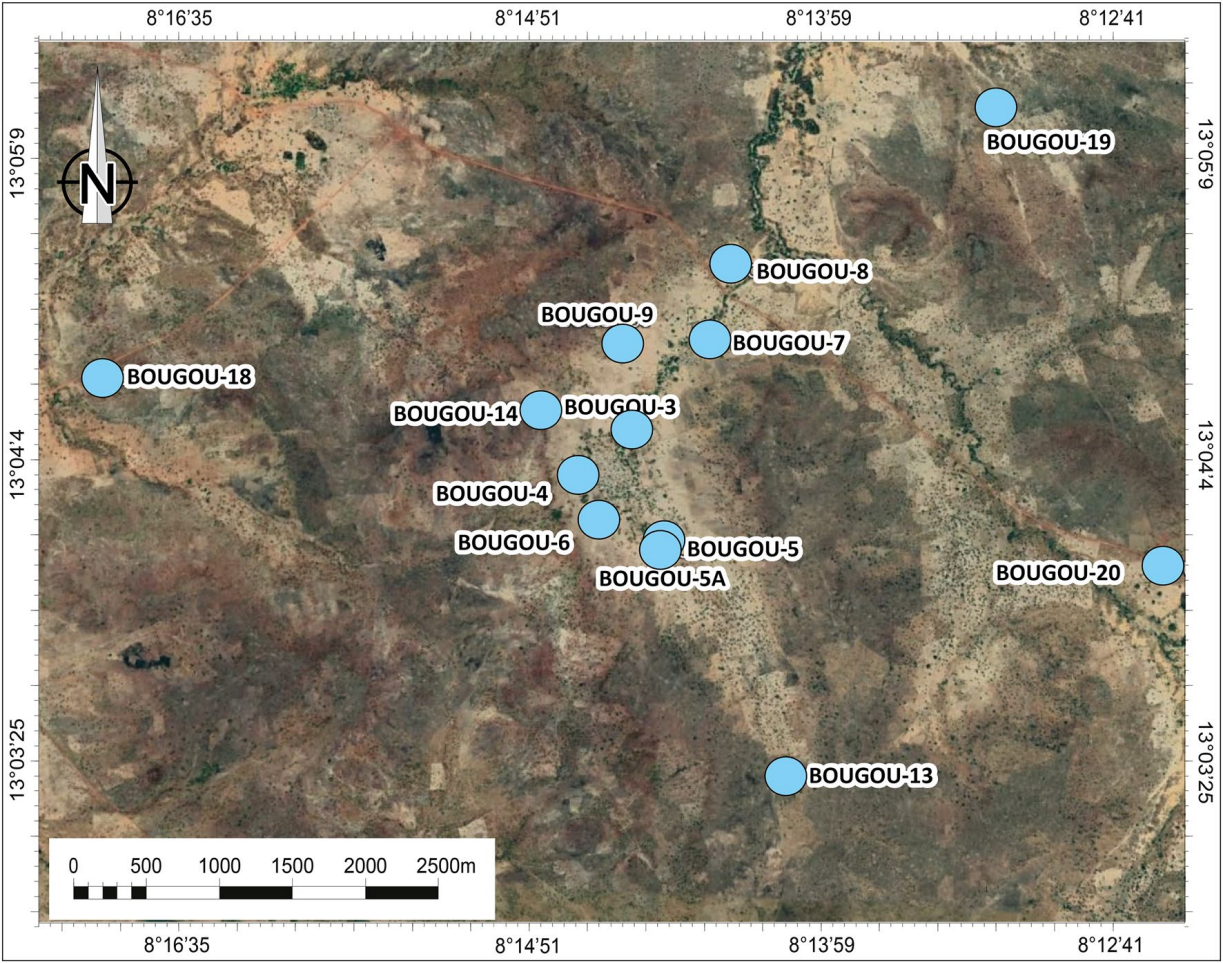
Position map of the thirteen wells used in this study, based on Google Earth satellite imagery of the Bourakebougou area (Mali) (https://earth.google.com/web/). The map was prepared using Adobe illustrator CS6 (Version 16.0.0, https://www.adobe.com/).

This study is based mainly on data collected during Hydroma’s 2018 exploration program. The study was performed on data from thirteen boreholes, notably Bougou-3, Bougou-4, Bougou-5, Bougou-5A, Bougou-6, Bougou-7, Bougou-8, Bougou-9, Bougou-13, Bougou-14, Bougou-18, Bougou-19, Bougou-20 (the position of the wells can be seen in Fig. 3). All the boreholes were recorded with electrical logs and core data. Twenty-one polished thin sections, Rock–Eval analyses and porosity measurement data were done on twenty-one samples from Bougou-20, Bougou-8, Bougou-18, and Bougou-7. In addition, calcimetric data performed by Exlog with a Bernard calcimeter on 129 samples were also used to identify the different carbonate levels along the deepest well. The Rock–Eval data was carried out at IFPEN and processed by applying the method of Pillot et al.^30^ to characterize and quantify carbonate contents in the reservoirs. Porosity measurements were also made at IFPEN, with helium being the gas used for porosity measurements. The polished thin sections were made by the Thin Section Laboratory (TSL) and were all made with impregnation by blue resin. An optical study was performed using a microscope for a petrographic and porosity study at the microscopic scale. An Alizarin coloration was done on a thin section according to the method described by Lindholm and Finkelman^31^. The logging data such as natural gamma-ray GN, bulk density RHOB and Neutron porosity NPHI were corrected, merged, and displayed using Petrel, Geolog and EasyTrace software. The raw data for the logs were provided by Hydroma and were performed by SemmLogging by using their gas chromatograph, gamma, Neutron, and density gamma Probes. In this study, the neutron log was calibrated in a Limestone matrix scale and the density and neutron were displayed in a standard limestone compatible scale.

The Borehole Imagery (ABI) performed by SemmLogging was also used to identify the fractures and karstic zones in the reservoirs. The Borehole Imagery data was obtained using a Borehole Tele Viewer with a fixed acoustic transducer and a rotating mirror to scan the borehole walls.

## Results

### Cores versus gas logging: where do we find H_2_?

**Figure 4:**
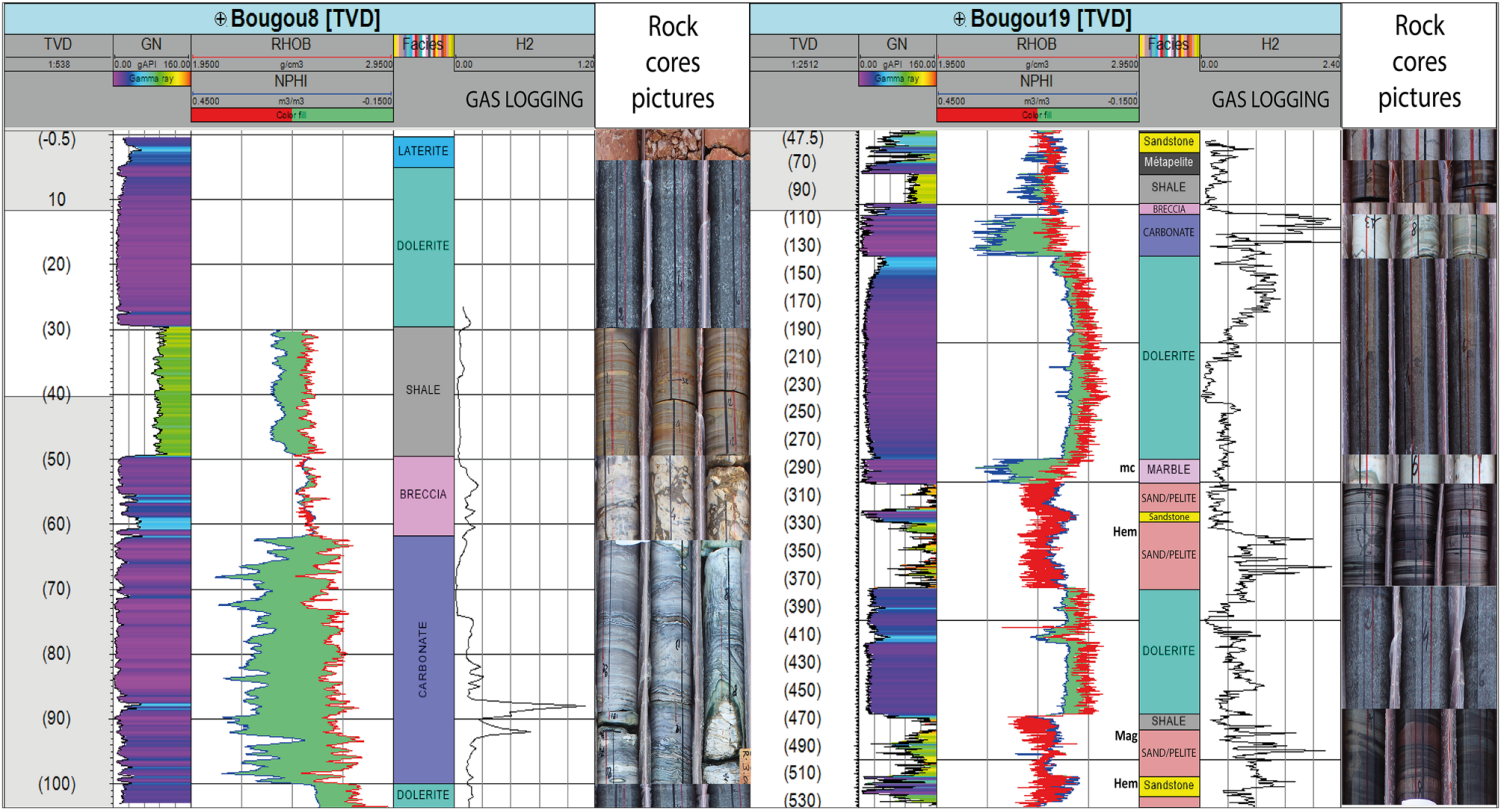
The different data used to identify and characterize the different formations and hydrogen accumulation zones between 0 and 530 m. Track 1: The true vertical depth, Track 2: The natural gamma-ray (GN), Track 3: The density (RHOB) and neutron porosity (NPHI) in limestone matrix scale, Track 4: The formation’s nature, Track 5: concentration of H_2_ from gas logging and Track 6: cores images. *mc* zone of intense contact metamorphism, *Mag* presence of magnetite, *Hem* presence of hematite.

To characterize the reservoirs of the Bourakebougou field, the hydrogen accumulation zones were first identified using gas logging data (Fig. 4). As can be seen in Fig. 4, on Bougou-19 (an example of a deep well), hydrogen accumulations were respectively found at 3 different depths: between 110 and 150 m, between 330 and 390 m and then between 465 and 520 m. The other two deeper accumulation zones identified by Prinzhofer et al.^7^ can be observed on the deepest well of the field (Fig. 2) respectively between 800 and 1040 m, and between 1130 and 1460 m (considering that an accumulation corresponds to a zone with a signature > 1 mol% of H_2_ in the mudlogging measurement on a thickness of more than 20 m). The well Bougou-8 presented in Fig. 4 is a shallow well, therefore only the first accumulation zone can be observed on it (Fig. 4). The first accumulation zone is observed on all the wells and corresponds to the one used to produce electricity. Correlations between several wells have shown that the shallowest reservoir is continuous and fairly horizontal with approximately the same thickness. This will be presented in a following paper (Maiga et al., in progress). At depth, the presence of hydrogen is also observed at the base of the series and in the fractured basement (Fig. 2). After identifying the various H_2_-rich zones**,** the geologic formations that are present at these zones were determined.

### The two types of H_2_ reservoirs identified

By calibrating the gas logging data and core images (Fig. 4), it appears that the accumulation of H_2_ occurred primarily within two types of formations: (1) carbonates and (2) sandstones. Figure 4 shows the different data that allowed this interpretation. From log responses and core descriptions, main lithologies have been identified. One can distinguish: (1) the clay formations, clearly identified on the core images and characterized in the logs by a high GN and low RHOB (Fig. 4); (2) lower down, the dolerites formations that are clearly identified on the core images and characterized in log by low GN. They have no porosity according to NPHI-RHOB logs and have high values of RHOB; (3) Below the dolerites, we find the carbonates. They are clearly identified in core images, and are characterized by a low GN, by a NPHI-RHOB response which either overlaps or shows a separation. As the logs are calibrated in a limestone matrix scale, the overlap of NPHI-RHOB in carbonates corresponds to calcite and the presence of separation between NPHI-RHOB with NPHI at left corresponds to dolomite; (4) Finally, the deeper sandstones that are well identified in core images are characterized in logs by a positive neutron density separation and by the density to the left of the neutron. In the sandstone reservoirs located at the base of the series, a clear increase in H_2_ in the iron-rich part is observed (Fig. 2). This raises the question of the possible oxidation of iron-rich levels as a source of H_2_. As this study is focused on the characterization of the H_2_ reservoirs, a detailed study of these iron-rich formations has not been carried out but the role of banded iron-rich formations in hydrogen generation has been discussed by Geymond et al.^32^. Regarding the basement, the constant increase in H_2_ that was observed suggests a probable hydrogen generation. Note that H_2_ generation related to radiolysis is not favoured within Neoproterozoic sediments because there is no H_2_ increase in the layers with the highest gamma-ray values (uranium-rich; Fig. 2).

### Characterization of the reservoirs

#### The main carbonate reservoir

**Figure 5:**
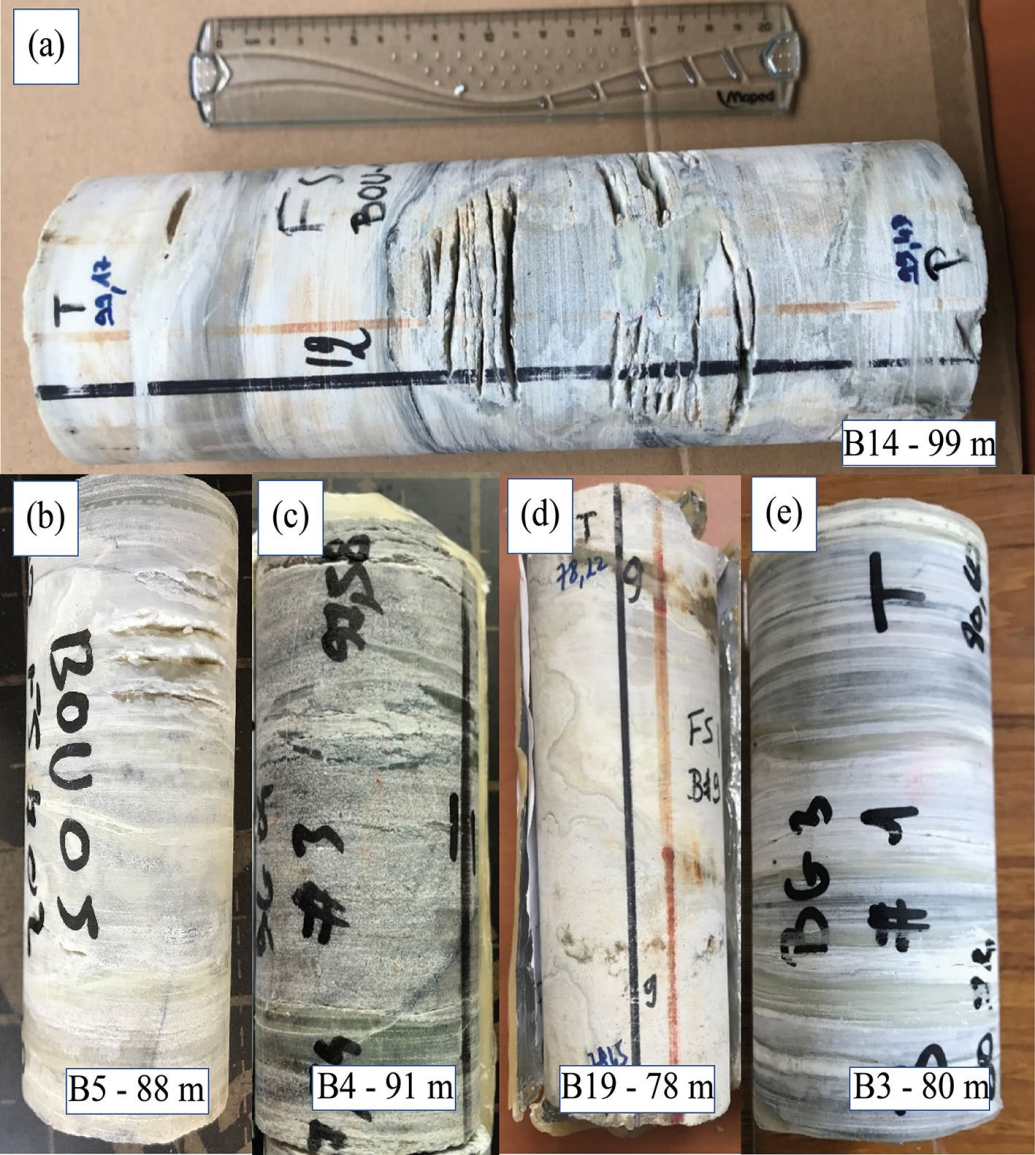
Macroscopic characterization of a karstic carbonate reservoir, cores are from Bougou-14, Bougou-5, Bougou-4, Bougou-19, and Bougou-3.

**Figure 6:**
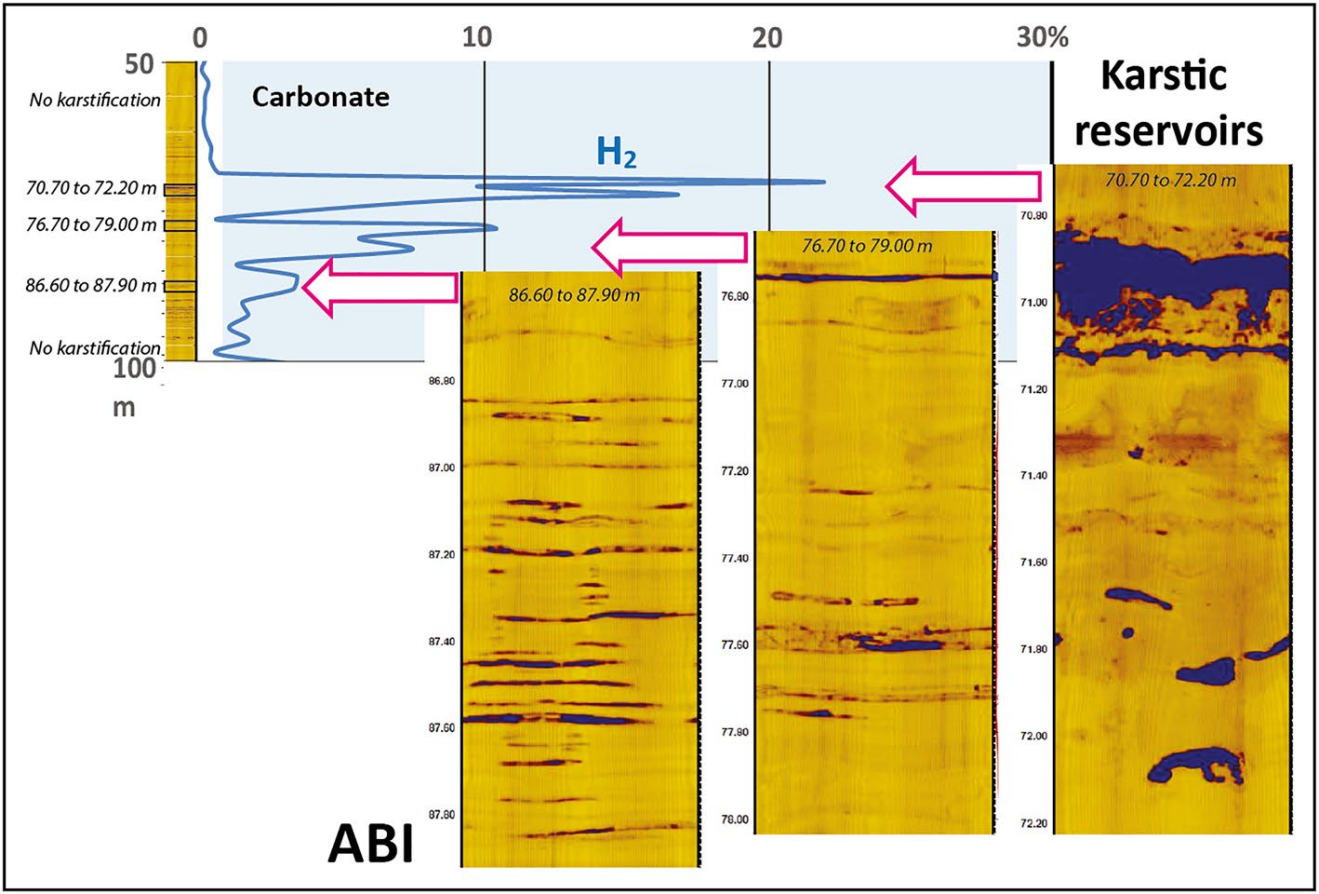
Borehole imagery versus gas logging showing the presence of hydrogen gas within the karstic system.

Macro analysis of sections from Bougou-14, Bougou-5, Bougou-4, Bougou-19 and Bougou-3 has shown that, in the upper dolomitic unit, carbonates are laminated (Fig. 5a–e) and that several carbonate zones are karstified and present cap carbonates nodules. The karstification is probably due to acid fluids related to CO_2_ degassing during the magmatic setting of the doleritic sills (the sourcing magma being rich in dissolved CO_2_). Indeed, karstification due to meteoritic fluids is not probable since the upper part of the carbonates is not karstified and the karstification only occurred close to the dolerite intrusions. The core sections close to the dolerite intrusion show cracks (Fig. 5) which are evidence of carbonate dissolution (thermo-karst). As Bougou-14 and Bougou-5 are about 2 km away from each other, there is indeed a large karstic system in this first reservoir zone. Marble zones are present near the bottom of the upper carbonate unit close to a doleritic sill. The marble zones have been shown in Fig. 9. These marbles can be interpreted as related to a contact metamorphism which occurred during the intrusion of the sill. The karstic nature and the presence of an aquifer system are also confirmed by Acoustical Borehole Imaging (ABI) (Fig. 6). As can be seen in Fig. 6, large voids are observable at different levels of the carbonate reservoirs.

Figure 6 shows that the hydrogen peaks correspond to these karstic zones. The comparison of the Acoustical Borehole Imagery and the gas logging data on the Bougou-6 well (Fig. 6) clearly shows that the hydrogen is contained in the karst (Fig. 6). This is the first time that the H_2_ accumulation in a karstified carbonate reservoir has been shown. Note that the amount of 30% shown during the gas logging is exceptionally high because there is always an important amount of air contamination in gas logging records. Compared to oil and gas reservoir systems, the accumulation patterns are therefore very similar.

#### Rock–eval and cross-plot analysis of the main carbonate reservoir

The results revealed that the karstified carbonate reservoir containing H_2_ is mainly a dolomitic carbonate. Of the 16 samples which were analysed, only 3 were primarily calcite. For example, two graphs corresponding to the results of samples taken from Bougou-3 and Bougou-13, which are about 2.5 km from each other, are shown in Supplementary Fig. S1. As the different types of carbonates can be identified by the temperature and shape of the peaks (notably the height and area of the peaks) according to Pillot et al.^30^, the red and orange peaks are close in both graphs (Supplementary Fig. S1) and appear in the temperature range of dolomites. This confirms the dolomitic nature of the karstified carbonate reservoir, and it was also confirmed by electric log analysis. By displaying the density (RHOB) as a function of the neutron porosity (NPHI) for the Bougou-13 well (Supplementary material Fig. S2), it was observed that all the plotted points were close to the dolomite line area with a slight offset. The calcimetric results (Supplementary Fig. S3) also confirm that the carbonates are predominantly dolomitic, and that the main reservoir is mainly dolomitic, which is consistent with the results of the rock–eval, logs and the microscopic analysis. Indeed, the dolomitic nature of the carbonate may be interesting for the H_2_ preservation as it is preserved in these carbonate rocks without reacting with carbonate carbon to produce methane.

#### Microscopic and petrophysical analysis of the main carbonate reservoir

**Figure 7:**
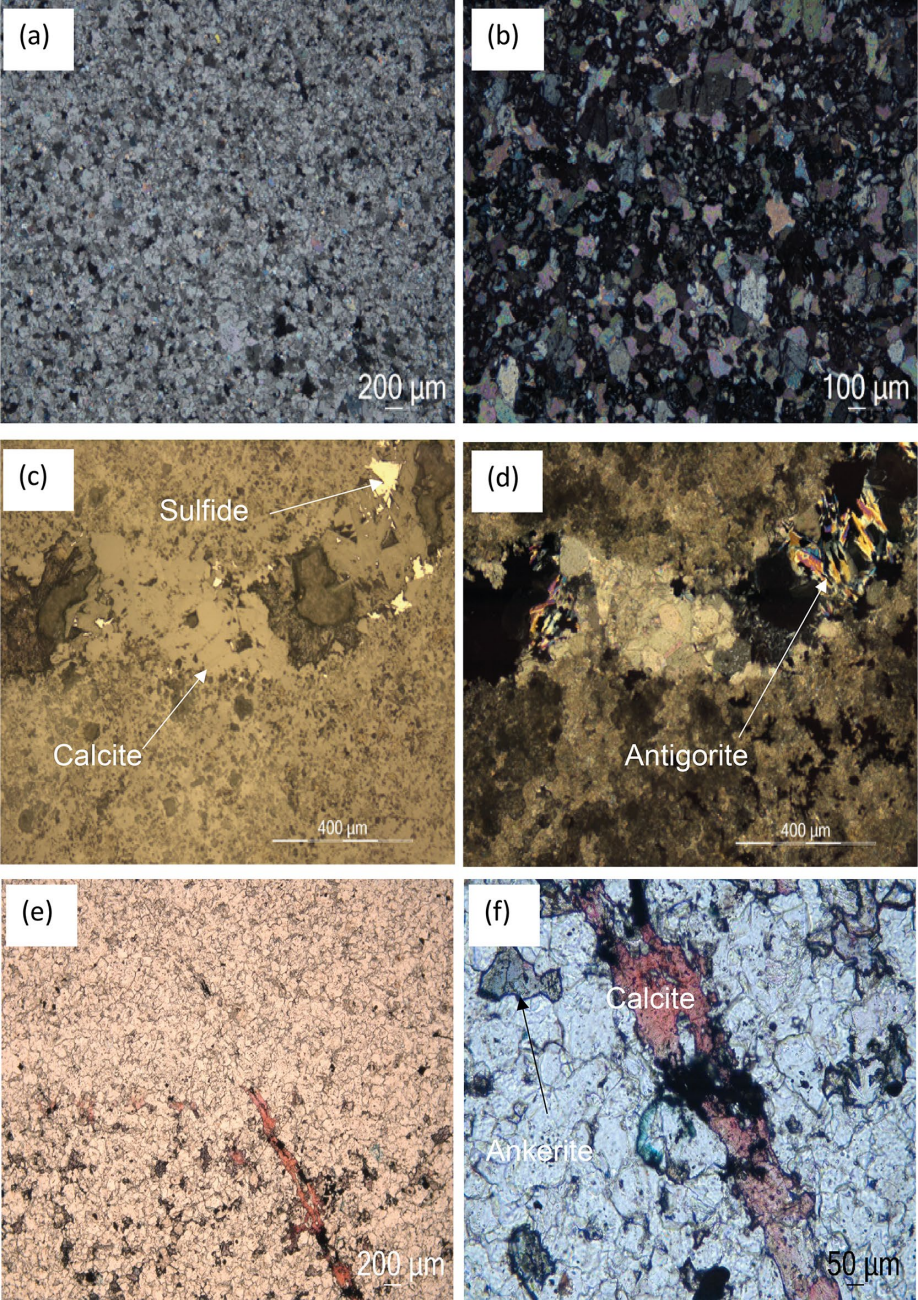
Carbonate reservoir observed by optical microscopy under (**a,b,d**) polarized and analysed light, (**c**) reflected and non-analysed light and (**d**) reflected and analysed light. (**a**) micro dolomite, (**b**) rhombohedral dolomite, (**c,d**) fracture with sulphide, calcite, and antigorite, (**e,f**) samples with alizarin coloration under unanalysed natural light.

The microscopic analysis confirmed the dolomitic nature of the carbonate reservoir at all scales (Fig. 7a–d). This is shown for instance when using alizarin colouring on a thin section of Bougou-8 (63, 48 m) (Fig. 7e and f). Alizarin is a chemical reagent that colours the calcite crystals in pinkish red, the ferriferous dolomites (ankerite) in very light blue, turquoise and/or green and it leaves the non-ferriferous dolomite colourless^31^. Two types of dolomites are observable on the thin sections. As shown in Fig. 7, a micro-dolomite with a xenotopic texture from Bougou-8 at 63.48 m (Fig. 7a) in addition to a dolomite matrix with rhombohedral minerals (idiotopic) from Bougou-20 at 96.48 m (Fig. 7b) are observable in polarized and analysed light. The presence of xenotopic texture with porphyrotopic fabrics indicates the replacing of a precursor limestone^33^. The thin section from Bougou-8 (68.09 m) shows the presence of sulphide (pyrrhotite) in optical microscopy under reflected and non-analysed light (Fig. 7c). In this same thin section, under reflected and analysed light (Fig. 7d), there is also evidence for fluid flow which we inferred from the presence of oxide, sulphide, and serpentine (antigorite). These minerals are observed in a fracture (Fig. 7c and d). Since the thin section has been impregnated with blue resin, this indicates that micro-porosity exists in these rocks. Based on the cross-cutting criteria, we observed a first dolomitized and fractured facies. After fracturing, a fluid circulation took place from which the three phases of calcite, antigorite, and sulphides precipitated. The chronology of these last three phases can be explained as follows: First, an engulfment of calcite by antigorite was observed in several places. This indicates that after the fluid circulation, the calcite precipitated first and then was replaced by antigorite. Finally, the sulphides developed and intersected the antigorites (Fig. 7c and d). On the coloured part of the thin section (Fig. 7e and f), a dolomitic matrix with calcite (red) (Fig. 7e and f) and ferriferous dolomite (ankerite) crystals (blue) can be distinguished (Fig. 7f). The presence of calcite-filled fractures and opaque crystals was also observed in Fig. 7e and f.

Helium porosity measurements were carried out on 10 samples collected from Bougou-8 and Bougou-20 wells in the dolomitic karstified zone (Supplementary Table S1). The porosities vary between 0.21 and 14.32%. This matrix heterogeneity of the carbonate matrix is consistent with the karstified nature of the dolomitic reservoir. However, porosity measurements on core plugs in a karstified reservoir are not representative of the whole formation which is controlled by a dual porosity between the porosity of the matrix and the large-scale porosity of the karstic voids.

#### Sandstone reservoirs

In Bougou-19 (one of the deep wells of the field, Fig. 4) and in Bougou-6 (the stratigraphic and deepest well of the field, Fig. 2), the H_2_-rich zones correspond to zones of iron-rich fine sandstone (Fig. 2) and sandstones with magnetite and hematite (Figs. 2 and 4). Some sandstones are also dolomitic-rich. In the borehole imagery, the deepest sandstones show numerous fractures in the lower part of the Bougou-6 well.

#### Microscopic and petrophysical analysis of the sandstone reservoirs

Microscopic observation of thin sections from samples from Bougou-19 and Bougou-20 provided more details on the sandstone reservoirs of the Bourakebougou field (Supplementary Fig. S4). Across a thin section from Bougou-19 well at a depth of 517 m, the porosity is filled with fine minerals (Supplementary Fig. S4). These fine minerals correspond to a serpentine which has recrystallized after an initial phase of partial dissolution of the quartz grains. Associated with these small minerals, secondary quartz recrystallized in the porosity (Supplementary Fig. S4). Disregarding the diagenetic siliceous cement, the grains have rounded and blunt shapes. This indicates transport by an erosive agent (wind or water). Helium porosity measurements were performed on 7 samples collected from Bougou-18 and Bougou-20 in the sandstone reservoir zones (Supplementary Table S2). These results show the homogeneity of the porosity values both at several depths and from several wells. The porosity ranges from 4.52 to 6.37%. This homogeneity is consistent with the matrix nature of the porosity of these sandstones. The porosity values shown in Supplementary Table S2 cannot be considered as a fully statistical dataset. These values only serve to screen the reservoir properties along the fairly homogeneous sandstone system (Fig. 2).

### Hydrogen presence identification by logging tools

**Figure 8:**
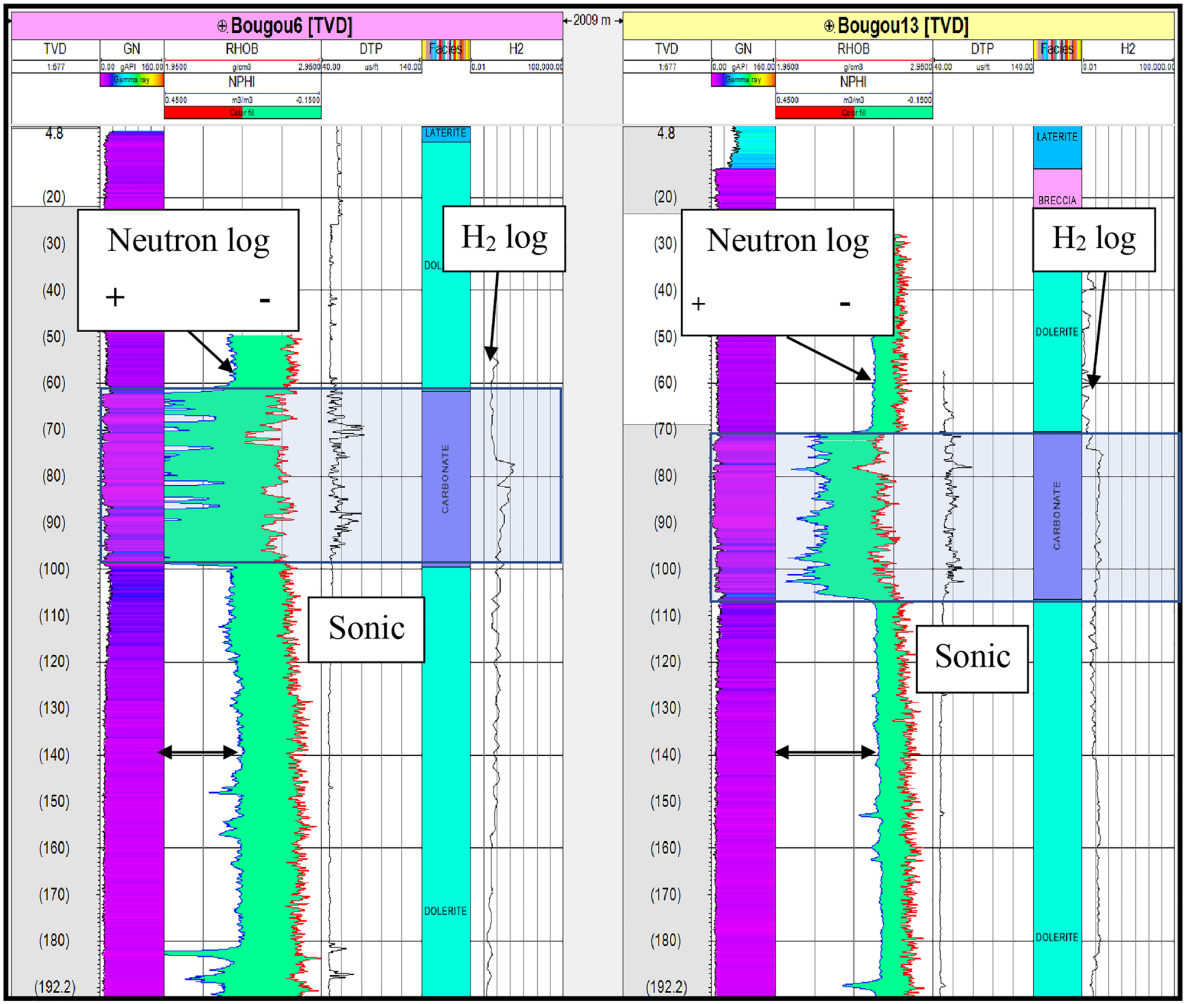
Neutron porosity comparison between a hydrogen-rich well (Bougou-6) and a hydrogen poor well (Bougou-13).

Cross-plot analysis of the electric logs in the uppermost carbonate reservoir zone has shown a shift with respect to the classical dolomitic line (Supplementary material Fig. S2). This shift reflects an abnormal increase in neutron porosity (NPHI). This is due either to the presence of shale or of hydrogen gas since they would affect the neutron values and then the interpreted porosity (increasing artefact). The neutron tool records the number of collisions between neutrons emitted by a source and the hydrogen atoms present in the rocks around the well. The neutron log is therefore a measure of the concentration of hydrogen atoms (sum of the H atoms in H_2_, H_2_O and OH) contained in both the pore fluids and in minerals. As in the first karstified reservoir of the Bourakebougou field, there is, indeed free hydrogen gas, it is then coherent that the neutron porosity becomes overexcited because of the quantity of hydrogen atoms counted. This can be directly observed by comparing a well with a high amount of hydrogen to another well with a low amount of hydrogen. In the case of the Bourakebougou field, the well with the highest concentration is the Bougou-6 well and the one with the lowest concentration is Bougou-13. By comparing these two wells (Fig. 8), it can be clearly observed that the Bougou-6 well shows higher values of neutron for the same formation compared to the Bougou-13 well. Therefore, the effect of the presence of H_2_ on the neutron tool is confirmed. Furthermore, from the sonic log (Fig. 8), a decrease of P-wave velocity in the main reservoir zone is also observed due to the presence of a gas phase. It is consistent with the previous results.

### Production test

**Figure 9:**
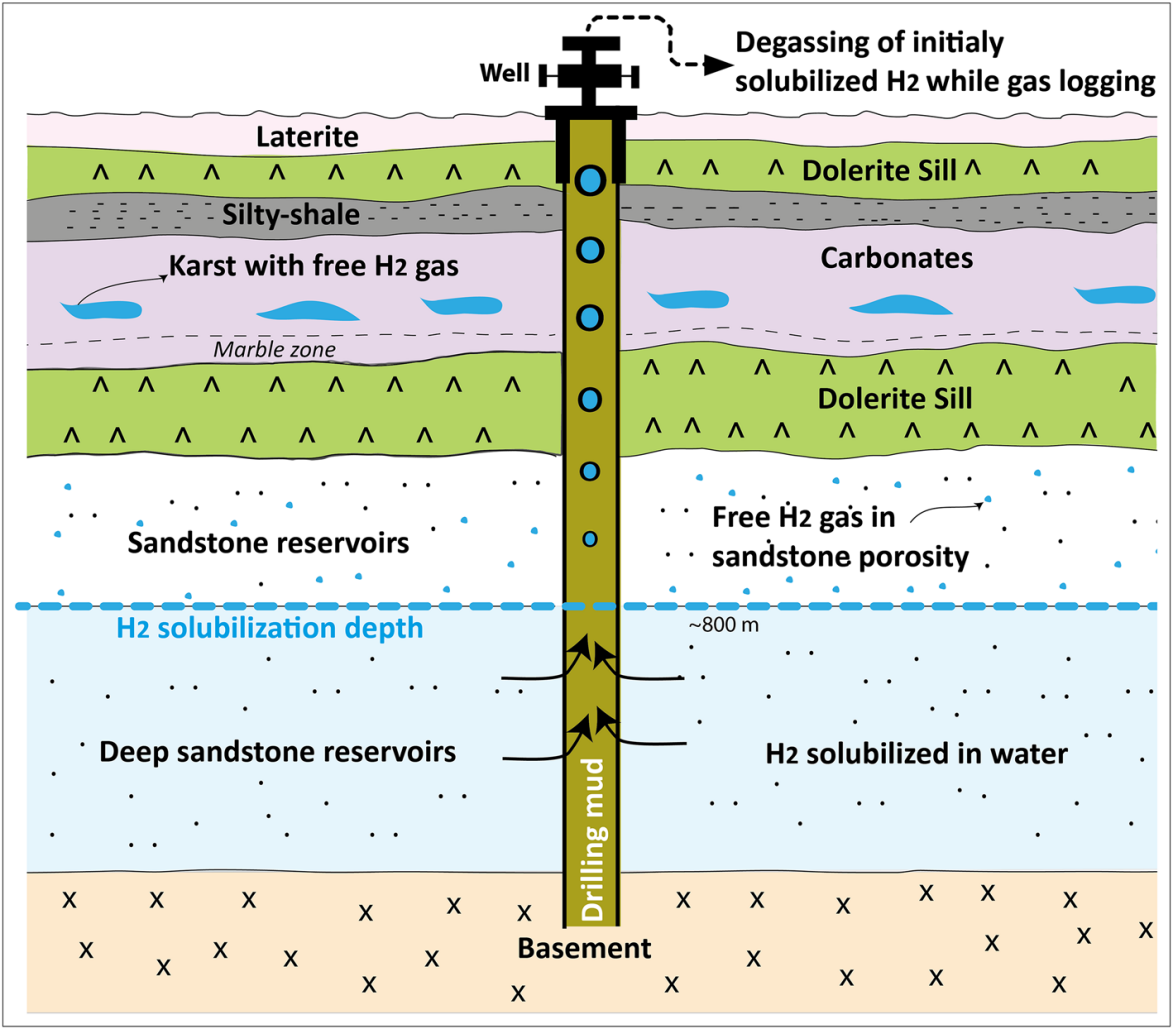
Synthetic diagram showing the presence of a free gas phase in the uppermost carbonate and sandstones reservoirs, with the gas mostly dissolved at depth especially in the deeper sandstone reservoirs then degassed in the rising drilling fluids. It also shows the dolerite intrusions and the marble location. The diagram was generated using Adobe illustrator CS6 (Version 16.0.0, https://www.adobe.com/).

The first H_2_ reservoir discovered when drilling for water has shown an approximate flow rate of 1500 m^3^ H_2_/day. It achieved this flow rate even though it was not a well that had been initially designed for a gas production and even though it had previously been cemented up before the test. By analysing the production data collected since the discovery of this historic well in 1987 up to the present, it was observed that after 11 years of operation to generate electricity, no pressure decline has been recorded as would be expected with a conventional oil and gas reservoir. To date, a pressure increase (from 4.5 to 5 bars) has been observed. This shows that the H_2_ accumulation is progressively recharged by a dynamic system. In the uppermost carbonate reservoir, water plays a role of barrier in the shallow aquifers below the water table in which H_2_ must necessarily migrate in gaseous form (because of its almost zero solubility at low pressure that makes diffusion less and less efficient toward the surface). This could explain why there is essentially the presence of free gas in very shallow reservoir, and no large accumulation of free gas discovered at depth. Knowing the temperature profile on the well that reached the basement (Bougou-6), using the pressure and temperature diagram containing the saturation curves according to Baranenko and Kirov^34^ (modified) (supplementary Fig. S5), it is possible to calculate the amount of hydrogen that can be stored in water versus depth. According to the available solubility measurements (cf. compiled from Baranenko and Kirov^34^, supplementary Fig. S5), hydrogen can be dissolved in very large quantities at depth. With pure water, it is only possible to store ~ 0.0214m^3^ H_2_ STP /m3 of water at the surface, whereas the possible stored volume of gas at the base of the well Bougou-6 is ~ 3m^3^ H_2_ STP /m^3^ of pure water. This implies that at a shallow depth and with a higher salinity in the wells, hydrogen should mostly be in a gas phase. This is consistent with the previous results on the presence of a gas phase in the uppermost main shallow reservoir. In the reference well Bougou-6, which is the richest in hydrogen and in which neutron anomalies have been highlighted in all the upper formations (Fig. 8), a neutron-density cross plot (supplementary Fig. S6) shows that anomalies in sandstone reservoirs are present from the uppermost reservoir down to a depth of 795 m. Below this depth, there are no neutron anomalies corresponding to H_2_-rich free gas, indicating that hydrogen is mainly dissolved in water but H_2_ is indeed also present at depth in important quantities as demonstrated by the gas logging results. This has been illustrated by a synthetic diagram in discussion (Fig. 9). The presence of free gas phase has been assumed only in the first three upper reservoirs out of the five that have been previously identified^7^.

## Discussion

The presence of oxides, sulphide, and serpentine in the carbonates is a result of past fluid circulation. The presence of mixed carbonates, particularly magnesian and ferrous carbonates (including ankerite), is also an evidence of fluid circulation. This circulation of hydrothermal fluids could be associated to contact metamorphism during the emplacement of the dolerite sills and the karstification of the carbonate could be as well related to acidic fluid circulation associated to magmatic degassing caused by the basic intrusions (Fig. 9). This could explain the characteristics of the main H_2_-bearing carbonate reservoir in Bourakebougou.

The results of gas logging, well imagery and production testing have shown that hydrogen at shallow depth is in the form of free gas in the porosity (mainly karstic voids in carbonates) and not trapped within the matrix of the carbonate. This suggests a conventional type of reservoir where it is indeed the voids that are filled with gas. However, unlike conventional oil and gas reservoirs, the accumulation processes are probably mainly controlled by a dynamic system. It is not just the volume of free gas present at time t that can be produced and that corresponds to the reserve in place. The potential of the field should not be considered simply as a static accumulation volume of free gas but also according to the degassing potential that is fed by a source of dissolved gas at depth and recharged progressively. The decrease of pressure towards the surface is responsible for a degassing process within the porosity at shallow depths. The residence time of H_2_ in the subsurface remains unknown so far. Because hydrogen is highly mobile and reactive, this residence time as a gas phase is expected to be relatively short but this would require further investigation and modelling. Potentially, one way to estimate the H_2_ recharge would be to monitor, before, during and after production, the quantity of dissolved H_2_ in the aquifers below the free gas zone. The free gas zone has been estimated to be about 800 m-thick, below H_2_ is considered to be mostly in dissolved form.

Indeed, in the uppermost carbonate and sandstones reservoirs, there is obviously the presence of a free gas phase whereas the gas may be mostly dissolved at depth especially in the deeper sandstone reservoirs and degassed in the rising drilling fluids during the gas logging process (Fig. 9).

At depth, in the Bougou-6 well, within the deep sandstone reservoir, there is a clear constant increase in H_2_ content in the iron-rich sandstones (‘BIF’-type) located at the base of the stratigraphic series (1300 to 1410 m, Fig. 2). The presence of iron-rich formations notably BIF are known to be potential sources to generate H_2_ by iron oxidation^32^. Indeed, BIFs have an oxidation potential when they have significant contents in Fe^II^. Here, the Fe^II^ is either completely oxidized to Fe^III^ or there is still Fe^II^ that is generating H_2_. In the sandstone reservoirs, the presence of magnetite, and serpentine (Supplementary Fig. S4) also indicates that at a period there was H_2_ generation in this zone (for example fayalite + water → magnetite + serpentine + H_2_). A recent study done by Geymond et al.^35^ also shows that magnetite may also generate H_2_ at low temperature. This also suggests a possible present-day generation of hydrogen. The question is: are we dealing with a genesis zone or a first deep reservoir in the Neoproterozoic sequence?

The logging results indicate that the neutron tool is affected by the presence of hydrogen when it is present as a gas phase but shows a non-anomalous behaviour when the hydrogen is present in a dissolved form. When characterizing the reservoir, the presence of an offset in the neutron density cross plot showing the dolomitic nature of the carbonates in the first reservoir was observed (Supplementary Fig. S2). In the oil and gas industry, there are two main hypotheses for interpreting this type of shift in a neutron-density cross plot. The first hypothesis is that the shift is due to the presence of a significant amount of clay in the reservoir and the second is that it is due to a gas effect. In this case study, this shift is linked to the presence of gas and not to the argilosity because the maximum value of the gamma-ray in the reservoir zone (35 gAPI) is not high enough to cause such a shift, whereas the gamma-ray interval varies between 0 and 250 gAPI for all the formations. Therefore, among the hypotheses, the second hypothesis is the one that is the most plausible. This interpretation is supported by the fact that there is a decrease in the P-wave velocity (through the sonic) indicating the presence of free gas in the reservoir zone (Fig. 8). As the main gas detected is hydrogen, it can be concluded that the neutron anomaly observed in the results is related to the presence of hydrogen. An analysis of the drilling mud parameters was done to observe if there was any hydroxyl component in the water or in the cores that could affect the neutron tool, but no evidence of a significant amount of hydroxyl was established.

## Conclusion

In the Bourakebougou field, the 25 wells that have been drilled have all discovered H_2_ in the upper carbonate reservoir. This suggests that this karstic reservoir is mostly connected in the whole area, but further correlation and interconnection studies will confirm this.

The shallowest main H_2_-bearing reservoir is primarily made of dolomitic carbonates (cap carbonates from the Neoproterozoic glacial episode). These predominantly dolomitic carbonates are extensively karstified and the largest accumulations of hydrogen are found within this karst system which extends over the field.

In the Bourakebougou field, the presence of H_2_ has also been observed at depth, notably in certain sandstone reservoirs and in the fractured basement. The hydrogen would be mainly in dissolved form at these depths and would outgas both in the upper sandstones and in the uppermost karstic carbonate reservoir because of its low solubility in water at shallow depths.

The shallow reservoirs would therefore represent the best potential zones for producing hydrogen, the free gas being retained by a very efficient seal which corresponds to dolerite intrusions.

Beyond gas logging, the neutron is the best candidate to attest and identify the presence of hydrogen. The presence of hydrogen generates a neutron anomaly by overestimating neutron porosity values.

The increase in pressure between the discovery period and today, knowing that the reservoir has been exploited to supply electricity for a decade, demonstrates that the hydrogen system is a dynamic system that is recharged while producing. Therefore, an assessment in the sense of a dynamic model should be considered to define an exact finite volume of gas in terms of reserve and in terms of rate of production.

Furthermore, the presence of serpentine and antigorite in the porosity of the dolomitic and sandstone reservoirs in addition to a clear increase in H_2_ in the iron-rich part of the sandstone reservoirs at the base of the series in Bougou-6 and in the fractured basement, raises the question of whether hydrogen generation is occurring in situ in these lithological units (iron oxidation being a classical process for natural H_2_ production).

## Supplementary Information

Supplementary Information.

## Data Availability

All data generated or analysed during this study are included in this published article and in its supplementary materials.
