# Supplementary material for: Characterization of the spontaneously recharging natural hydrogen reservoirs of Bourakebougou in Mali
Source: Sci Rep. 2023 Jul 22;13:11876. doi: 10.1038/s41598-023-38977-y (PMC10363119; doi:10.1038/s41598-023-38977-y)
Supplement: Supplementary file 1 — Supplementary Information. [file 41598_2023_38977_MOESM1_ESM.pdf]

Characterization of the Bourakebougou Natural Hydrogen Reservoirs in Mali

Scientific Reports,

Maiga, Omar[1] – omar.maiga@ifpen.fr – IFPEN-IFP-SCHOOL, Rueil-Malmaison, France–Corresponding author*, Deville, Eric[1], Laval, Jérome[1], Prinzhofer, Alain[2]; Diallo, Aliou Boubacar[3], [1]French Institute of Petroleum and New Energies, IFP-School, Rueil-Malmaison, France; [2]GEO4U, Rio de Janeiro, Brazil; [3]HYDROMA INC, Montréal, Québec, Canada.

Rock-Eval analysis

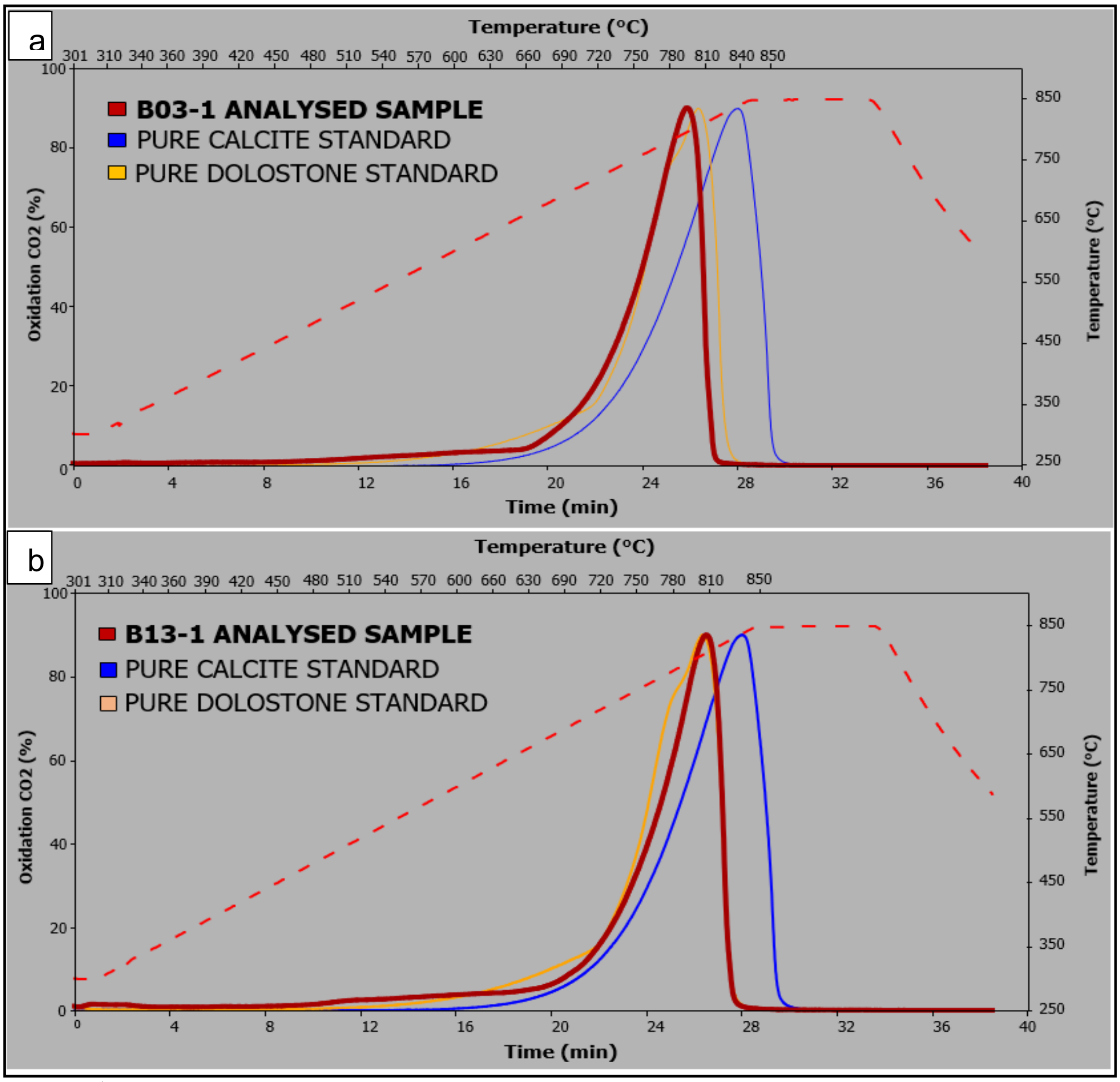

Fig. S1. (a) Results obtained on sample from Bougou-3's karstified carbonate reservoir and on two different pure carbonate standards: dolomite and calcite (b) Results obtained on sample from Bougou-13's karstified carbonate reservoir and on two different pure carbonates standard: Pure dolomite and pure calcite. The graph shows the monitoring of the CO_2 flux emitted by progressive oxidizing thermal decomposition during a programmed heating of the carbonate samples as a function of temperature and time. Each graph shows 3 curves (Red, orange, and blue), the red curve corresponds to the samples analysed named B03-1 (SM-1a) and B13-1 (SM-1b) and the others (Orange and Blue) to pure standard carbonate type used for the identification (SM-1a, b).

Characterization of the Bourakebougou Natural Hydrogen Reservoirs in Mali

Scientific reports,

Maiga, Omar[1] – omar.maiga@ifpen.fr – IFPEN-IFP-SCHOOL, Rueil-Malmaison, France–Corresponding author*, Deville, Eric[1], Laval, Jérome[1], Prinzhofer, Alain[2]; Diallo, Aliou Boubacar[3], [1]French Institute of Petroleum and New Energies, IFP-School, Rueil-Malmaison, France; [2]GEO4U, Rio de Janeiro, Brazil; [3]HYDROMA INC, Montréal, Québec, Canada.

Cross plot analysis

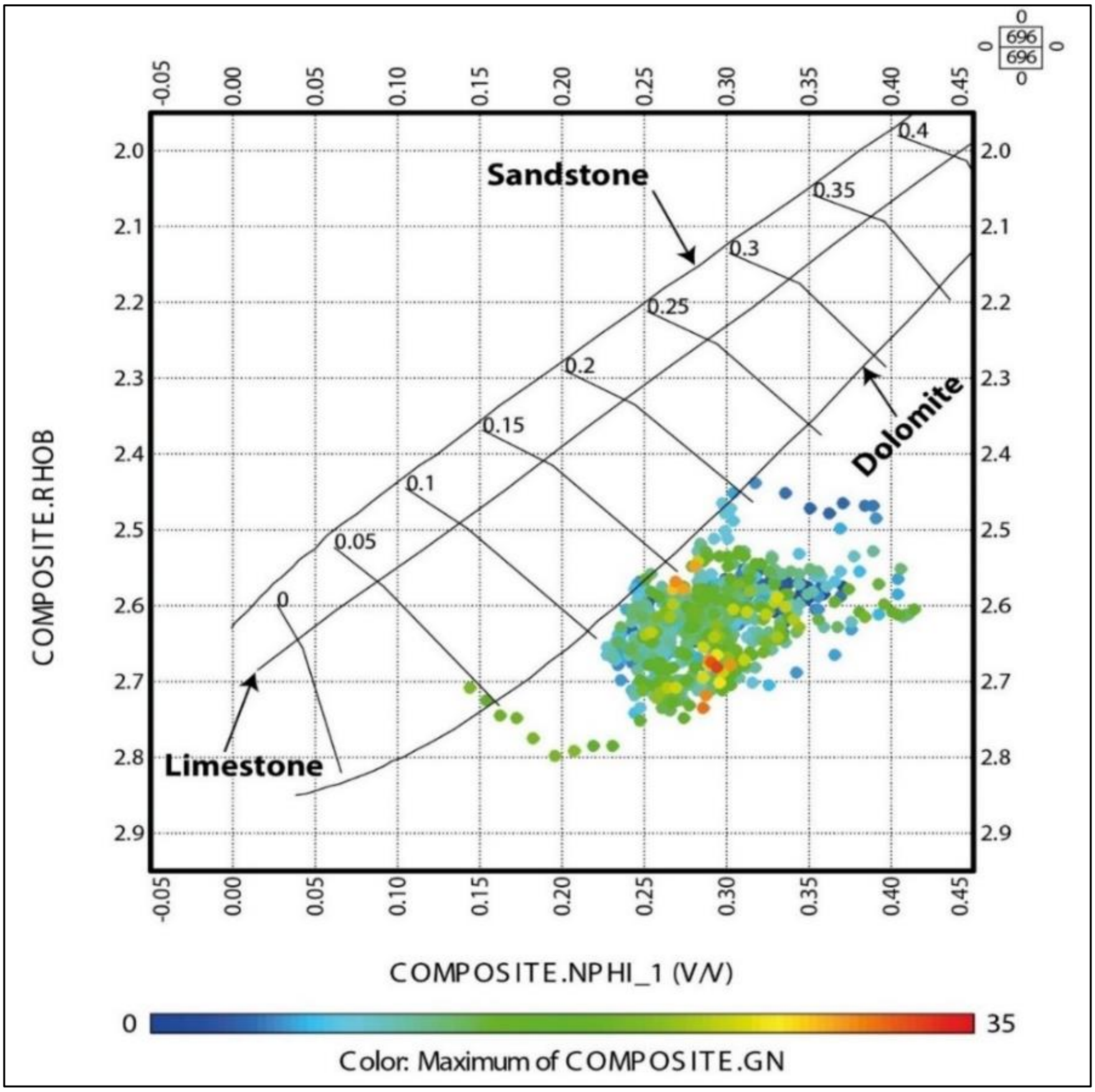

Fig. S2. Cross plot of density log (RHOB) versus neutron porosity log (NPHI) for the Bougou-13 well, the colour table shows the shale contents of the formation.

Characterization of the Bourakebougou Natural Hydrogen Reservoirs in Mali

Scientific Reports,

Maiga, Omar[1] – omar.maiga@ifpen.fr – IFPEN-IFP-SCHOOL, Rueil-Malmaison, France–Corresponding author*, Deville, Eric[1], Laval, Jérome[1], Prinzhofer, Alain[2]; Diallo, Aliou Boubacar[3], [1]French Institute of Petroleum and New Energies**,** IFP-School, Rueil-Malmaison, France; [2]GEO4U, Rio de Janeiro, Brazil; [3]HYDROMA INC, Montréal, Québec, Canada.

Calcimetric analysis

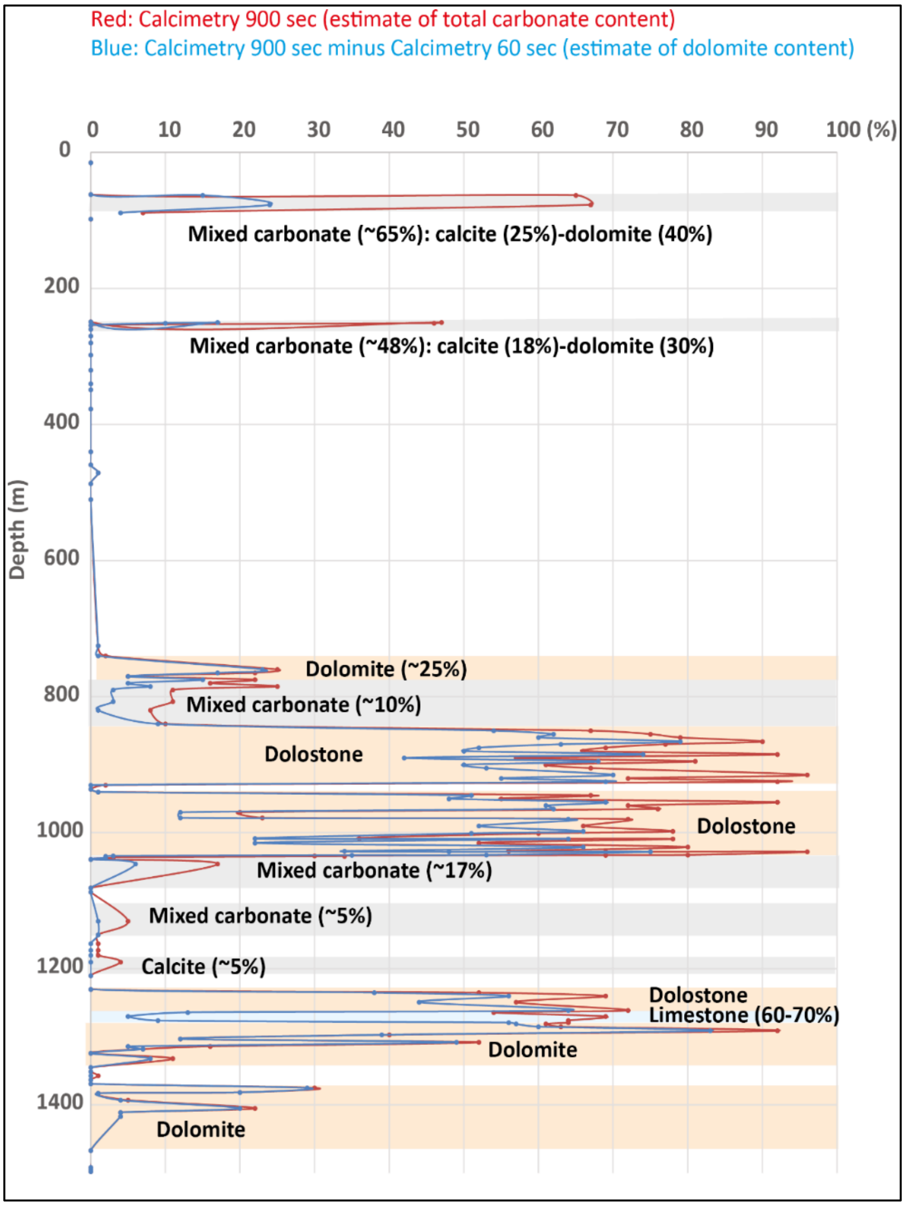

Fig. S3. Results of the calcimetric analysis for hundred and twenty-nine samples along the well that reached the basement (Bougou-6). The red curve shows the estimate of the total carbonate content dissolved after 900 seconds of acid attack. The blue curve corresponds to the total carbonate content dissolved after 900 seconds of acid attack minus the carbonate content dissolved after 60 seconds of acid attack, which provides an estimate of dolomite content.

Characterization of the Bourakebougou Natural Hydrogen Reservoirs in Mali

Scientific Reports,

Maiga, Omar[1] – omar.maiga@ifpen.fr – IFPEN-IFP-SCHOOL, Rueil-Malmaison, France–Corresponding author*, Deville, Eric[1], Laval, Jérome[1], Prinzhofer, Alain[2]; Diallo, Aliou Boubacar[3], [1]French Institute of Petroleum and New Energies, IFP-School, Rueil-Malmaison, France; [2]GEO4U, Rio de Janeiro, Brazil; [3]HYDROMA INC, Montréal, Québec, Canada.

Petrophysics analysis of the carbonate reservoirs

Well ID	Depths (m)	Porosity (%)
Bougou-8	94.57	1.2
Bougou-8	73.49	1
Bougou-8	68.09	14.3
Bougou-8	63.48	0.2
Bougou-8	83.89	0.3
Bougou-20	93.50	0.8
Bougou-20	87.16	10.7
Bougou-20	96.48	0.7
Bougou-20	62.99	4.6
Bougou-20	64.60	8.9

Table S1. Porosity measurement values on 10 samples from Bougou-8 and Bougou-20

Characterization of the Bourakebougou Natural Hydrogen Reservoirs in Mali

Scientific Reports,

Maiga, Omar[1] – omar.maiga@ifpen.fr – IFPEN-IFP-SCHOOL, Rueil-Malmaison, France–Corresponding author*, Deville, Eric[1], Laval, Jérome[1], Prinzhofer, Alain[2]; Diallo, Aliou Boubacar[3], [1]French Institute of Petroleum and New Energies, IFP-School, Rueil-Malmaison, France; [2]GEO4U, Rio de Janeiro, Brazil; [3]HYDROMA INC, Montréal, Québec, Canada.

Microscopic analysis of the sandstone reservoirs

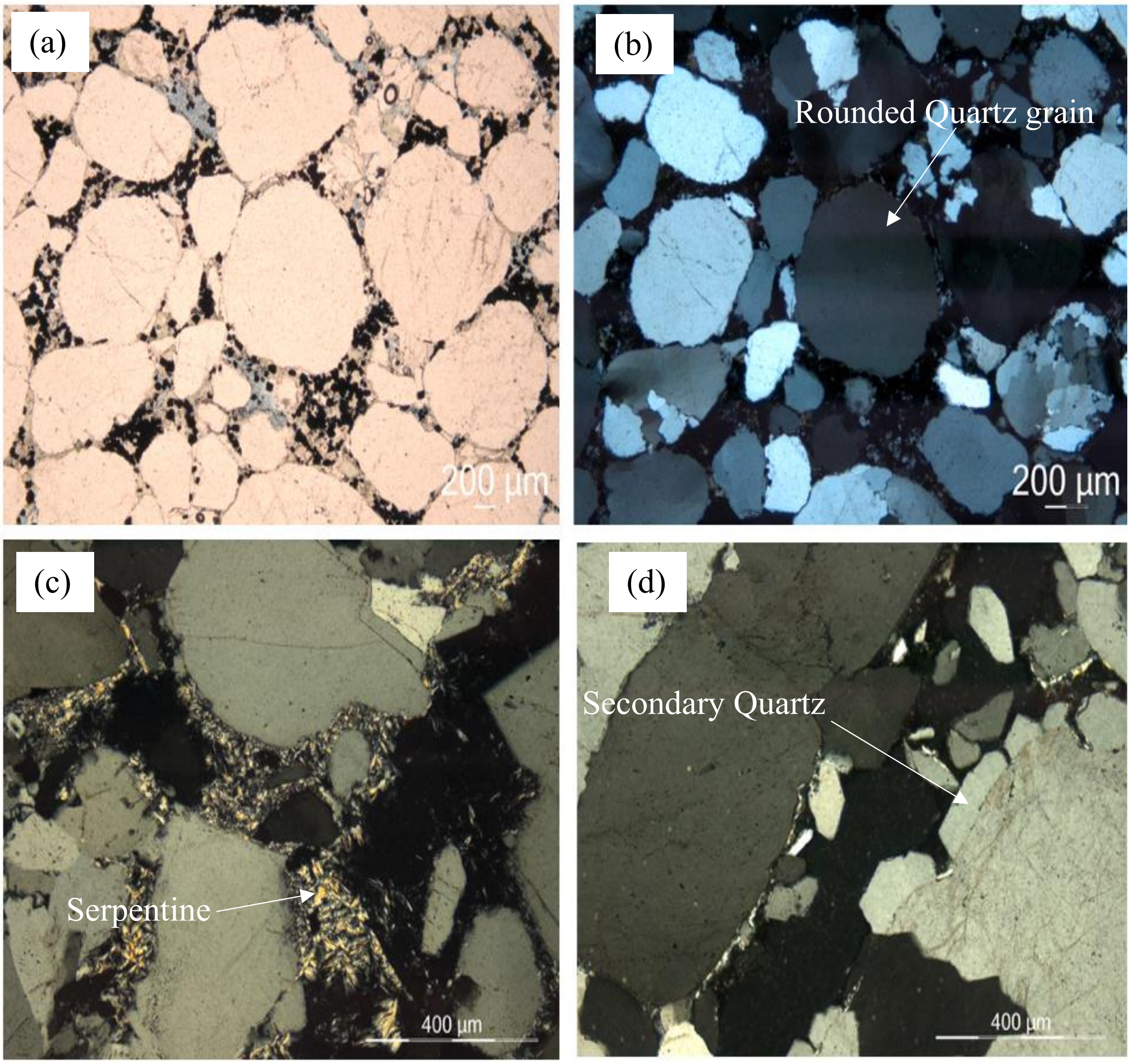

Fig. S4. Sandstone reservoir observed by optical microscopy under (a) polarized and non-analysed light, (b, c, d) polarized and analysed light. (a) and (b) are samples from Bougou-20, 317 m, (c) and (d) are samples from Bougou-19, 517 m.

Characterization of the Bourakebougou Natural Hydrogen Reservoirs in Mali

Scientific Reports,

Maiga, Omar[1] – omar.maiga@ifpen.fr – IFPEN-IFP-SCHOOL, Rueil-Malmaison, France–Corresponding author*, Deville, Eric[1], Laval, Jérome[1], Prinzhofer, Alain[2]; Diallo, Aliou Boubacar[3], [1]French Institute of Petroleum and New Energies, IFP-School, Rueil-Malmaison, France; [2]GEO4U, Rio de Janeiro, Brazil; [3]HYDROMA INC, Montréal, Québec, Canada.

Petrophysics analysis of the sandstone reservoirs

Well ID	Depths (m)	Porosity (%)
Bougou-18	443,54	4.5
Bougou-18	471,51	5.2
Bougou-20	541,85	6.3
Bougou-20	500,59	6.4
Bougou-20	473,17	4.6
Bougou-20	317,25	5.8
Bougou-20	326,10	5.9

Table S2. Porosity measurement values on 7 samples from Bougou-18 and Bougou-20

Characterization of the Bourakebougou Natural Hydrogen Reservoirs in Mali

Scientific Reports,

Maiga, Omar[1] – omar.maiga@ifpen.fr – IFPEN-IFP-SCHOOL, Rueil-Malmaison, France–Corresponding author*, Deville, Eric[1], Laval, Jérome[1], Prinzhofer, Alain[2]; Diallo, Aliou Boubacar[3], [1]French Institute of Petroleum and New Energies, IFP-School, Rueil-Malmaison, France; [2]GEO4U, Rio de Janeiro, Brazil; [3]HYDROMA INC, Montréal, Québec, Canada.

Production Test: Hydrogen storage capacity at shallow depth and at depth

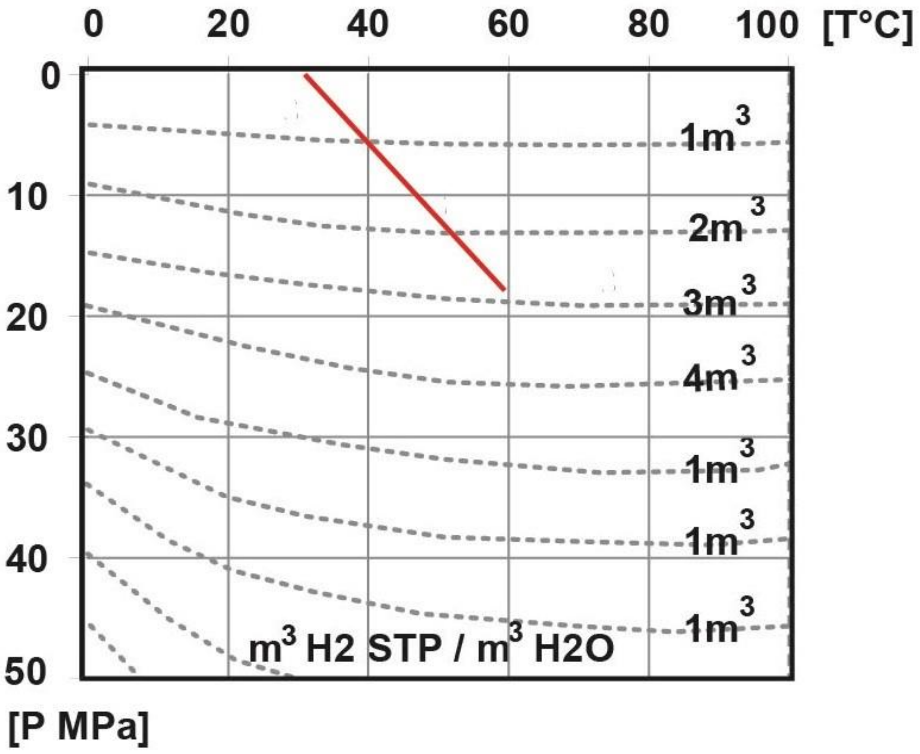

Fig. S5. Pressure versus Temperature diagram compiled from Baranenko et al., (1989). The red line corresponds to the temperature and pressure profile of the deepest well (Bougou-6). The storage capacity of hydrogen is ~0.0214m^3 H_2 STP /m3 H_2O at atmospheric pressure, whereas the possible stored volume of gas at the base of the well Bougou-6 is ~3m^3 H_2 STP /m^3 pure H_2O.

Characterization of the Bourakebougou Natural Hydrogen Reservoirs in Mali

Scientific Reports,

Maiga, Omar[1] – omar.maiga@ifpen.fr – IFPEN-IFP-SCHOOL, Rueil-Malmaison, France–Corresponding author*, Deville, Eric[1], Laval, Jérome[1], Prinzhofer, Alain[2]; Diallo, Aliou Boubacar[3], [1]French Institute of Petroleum and New Energies, IFP-School, Rueil-Malmaison, France; [2]GEO4U, Rio de Janeiro, Brazil; [3]HYDROMA INC, Montréal, Québec, Canada.

Cross plot analysis : Free H_2-gas in upper reservoirs and dissolved hydrogen at depth

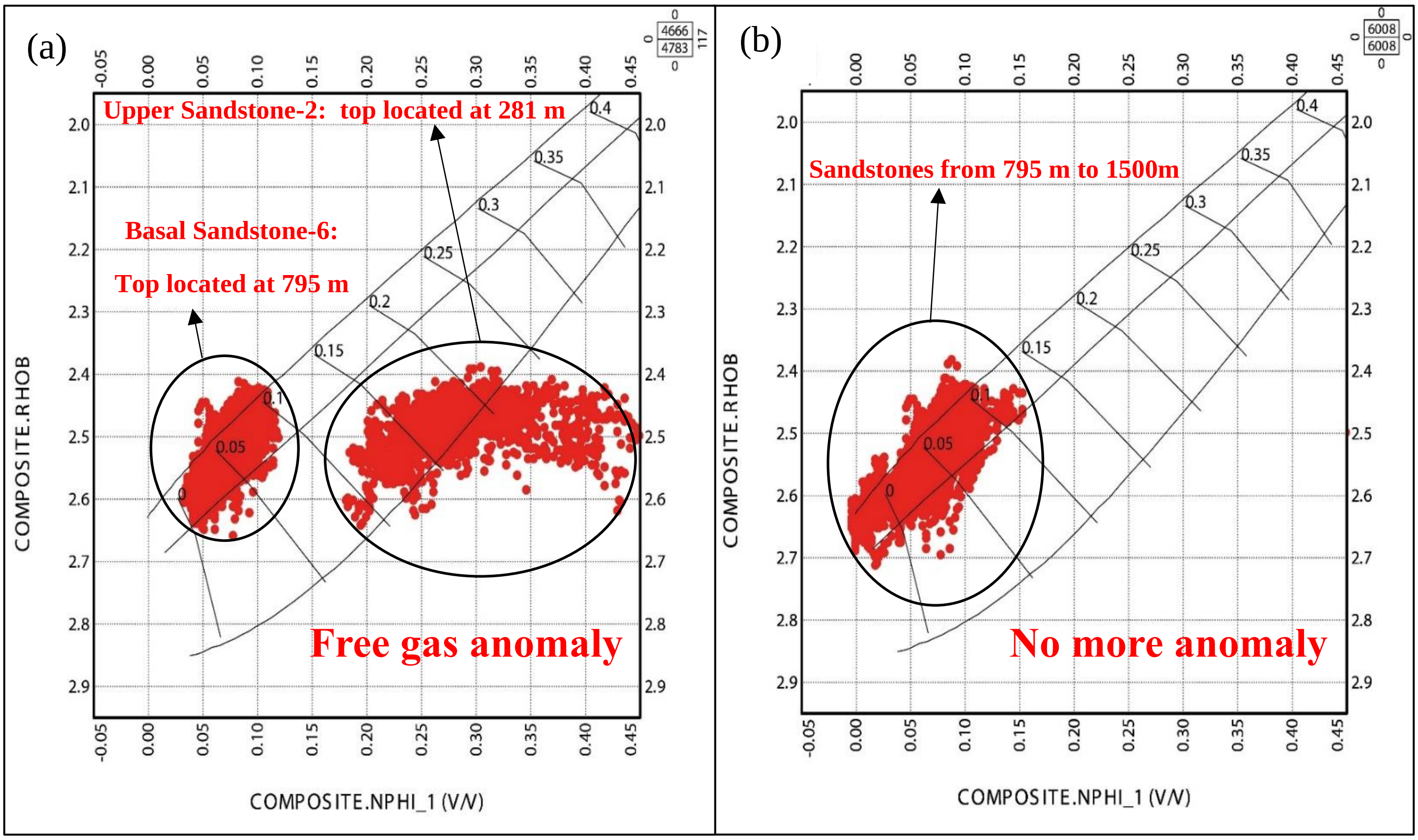

Fig. S6 Cross plot of density log (RHOB) versus neutron porosity log (NPHI) for the Bougou-6 well. (a) Sandstone reservoir between 281 m to 795 m Interval (B) Sandstone reservoir between 795 m to 1500 m Interval
